# Detection of different variants of SARS-CoV-2 RNA (genome) on inanimate surfaces in high-touch public environmental surfaces

**DOI:** 10.1038/s41598-023-40342-y

**Published:** 2023-08-11

**Authors:** Zahra Noorimotlagh, Seyyed Abbas Mirzaee, Faezeh Seif, Mojtaba Kalantar, Tayebeh Roghani, Seyed Ali Mousavi, Azam Honarmandpour

**Affiliations:** 1 Health and Environment Research Center, Ilam University of Medical Sciences, Ilam, Iran; 2 Department of Environmental Health Engineering, Faculty of Health, Ilam University of Medical Sciences, Ilam, Iran; 3 Department of Basic Sciences, Shoushtar Faculty of Medical Sciences, Shoushtar, Iran; 4 Department of Public Health, Shoushtar Faculty of Medical Science, Shoushtar, Iran; 5 Department of Midwifery, Shoushtar Faculty of Medical Sciences, Shoushtar, Iran

**Keywords:** Environmental sciences, Diseases

## Abstract

Severe acute respiratory syndrome coronavirus 2 (SARS-CoV-2) disease started in late 2019 and still continues as a global pandemic, spreading among people around the world. There is limited knowledge about the role of contaminated environmental surfaces, especially high-touch public surfaces, in the transmission of the disease. The objective of the present investigation was detection of different variants (Delta, UK, and Omicron) of SARS-CoV-2 RNA (genome) on inanimate surfaces in high-touch public environmental surfaces in different seasons. Automated teller machines of banks (ATM), point-of-sale (POS) machine, gas station pump nozzles, and escalator handrails of malls were selected as high-touch environmental surfaces in public places. Overall, 75 samples were collected from these places and examined for the presence of SARS-CoV-2 RNA (genome), and 21 samples (28%) were positive. Although the role of fomite transmission of COVID-19 is understood, more studies should be conducted to determine the virus survival rate as well as the required efforts to prevent the spread of SARS-CoV-2 such as frequent cleaning and the use of efficient disinfectants on environmental surfaces, especially high-touch public places. In conclusion, the results address the importance of touching contaminated inanimate objects as well as transmission through environmental surfaces, and they could be used to establish an effective protocol to prevent indirect environmental transmission of SARS-CoV-2, slow down the spread of the virus, and reduce the risk of infection.

## Introduction

The global pandemic situation of severe acute respiratory syndrome coronavirus 2 (SARS-CoV-2) started in late 2019 in Wuhan city, China, and it was called COVID-19 pandemic by World Health Organization (WHO) on March 12, 2020. The novel human coronavirus belongs to the Betacoronavirus genus and there are seven human coronaviruses (HCoVs) detected until today including HCoV‐229E, HCoV‐NL63, HCoV‐OC43, HCoV‐HKU1, severe acute respiratory syndrome coronavirus (SARS‐CoV), Middle East respiratory syndrome coronavirus (MERS‐CoV), and SARS‐CoV‐2. Among these human HCoVs, four of them including HCoV‐OC43, HCoV‐229E, HCoV‐HKU1, and HCoV‐NL63 usually lead to mild to moderate respiratory diseases. SARS-CoV-2 has the third known zoonotic virus, which was spread after SARS-CoV-1 and MERS^1–4^. It is reported that SARS-CoV-2 encodes more than seven accessory proteins (ORF3a, ORF3b, ORF6, ORF7a, ORF7b, ORF8, and ORF9b), and it should be mentioned that this virus encodes ORF9c and ORF10, which play a role in the immune evasion process^5^.

Based on the situation report of WHO, until November 9, 2022, there have been 630,387,858 confirmed cases of COVID-19 (SARS-CoV-2) including 6,583,163 deaths worldwide. It is worth noting that as of November 1, 2022, a total of 12,861,382,558 vaccine doses have been administrated. The pandemic situation led to use of environmental surface disinfectants and alcohol–based hand gel, as well as social distancing as preventive measures to prevent viral transmission through fomites^6–8^. With this information in mind, this disease is still spreading and affecting people around the world.

One of the most important traits of COVID-19 is its multi-route transmission potential. Therefore, an important prerequisite for effective and complete prevention and control of the disease is understanding the different modes and mechanisms of transmission. There are various routes and mechanisms of transmission for COVID-19, which are categorized into direct and indirect routes. The direct routes include droplet and bioaerosol emission pathways and human-to-human (person-to-person) mechanisms. In the indirect routes, the contaminated objects, environmental surfaces, high-touch public places, and airborne routes of SARS-CoV-2 are of utmost importance^9–11^.

Amongst the transmission routes, transmission by contaminated objects and environmental/inanimate surfaces, especially high-touch environmental surfaces in public places due to the potentially contain a high load of SARS-CoV-2 genome is utmost importance. Therefore, detection and determination of the SARS-CoV-2 genome in high-touch environmental surfaces could be an early warning sign to prevent and control COVID-19 disease^1,12–19^. On the other hand, there are several new variants of COVID-19 that have been reported with distinct abilities to survive on environmental surfaces under different meteorological conditions, i.e., relative humidity (RH) and temperature^1,6,20,21^.

Therefore, the aim of the present work, as the first study focused on the different variants of SARS-CoV-2 RNA (genome) on inanimate surfaces, was determination and detection of different variants of SARS-CoV-2 RNA (genome) on high-touch public and inanimate environmental surfaces under different seasonal conditions.

## Materials and method

### Study design and sample size

The current research is a part of a cross-sectional study that was conducted on 75 samples with the aim of “investigating the level of environmental contamination in public places in Shoushtar city in terms of presence of different variants of COVID-19 in different seasons of the year carried in a period from 2021-3-10 to 2022-3-10”.

### Approval statement and sampling site description and approval of ethics code statement.

The present research was supported and approved by Shoushtar Faculty of Medical Sciences (The code of ethics IR.Shoushtar.Rec.1402.010). All methods were performed in accordance with the relevant guidelines and regulations^22,22,23,23,24^. Shoushtar city is one of the most populated and touristic cities of Khuzestan province. It is located in the southwest of the Islamic Republic of Iran and has a population of about 225,000 people. Sampling was done to detect SARS-CoV-2 in the surrounding surfaces in crowded areas of the city at three stages that were simultaneous with the peaks of the UK, the Delta, and the Omicron variants.

### Sample collection

The sampling locations included two large shopping centers, 10 automated teller machines (ATM) of banks, four gas station pump nozzles, and five mobile point-of-sale (POS) machines. A total of 75 samples were taken in three seasons: spring, summer and autumn, which coincided with the arrival of the above-mentioned variants. Sampling was done at three points in time (simultaneously with confirmation of diagnosis of the new variant in the region by sending random samples to the National Reference Laboratory). Due to the small number of large shopping centers and fuel stations in the city, sampling was done from all these centers (census), but in the case of banks and mobile POS machines, sampling was done randomly (simple random sampling) from all parts of the city with more emphasis on crowded places with higher density. Swab sampling was done in the same way as in the study of Faezeh et al.^1^. Briefly, swab was moistened with Viral Transport Medium (VTM). Using a separate sterile collection swab and VTM for each surface, the swab was applied to an area measuring 10 × 10 cm, then the swab was returned to the VTM. After sample collection, they were transported to the reference laboratory in cool boxes (ensure the respect of the cold chain using the thermometer) while employing standard precautions and proper cold chain. In order to control the bias, all the samples were obtained at a certain time of the day (5–7 PM). The staff carrying out the sampling were fully clothed in gloves and face shields. In order to check the relationship between the level of contamination and climatic variables, temperature (°C) and relative humidity (%) were also monitored and recorded during environmental surface sampling using the portable weather station (Kimo).

### Viral genome extraction

The viral RNA was extracted using a Roje Technologies kit (Pishgam, Iran) and was stored in a deep freezer (− 70 °C). Subsequently, a real-time (RT)-PCR test was used to determine the presence of SARS-CoV-2 RNA in the collected environmental surface samples.

### Detection of virus infections by RT-PCR

According to the manufacturer’s instructions (Pishtaz Teb Zaman, Iran)*,* 5 μL extracted RNA was added to 15 μL of One-Step qRT-PCR master mix supplemented with a modified RdRp and N gene primer/probe sets. All real-time RT-PCR experiments were carried out by the Applied Biosystems Step One plus RT-PCR System. In the kit that was used, complementary DNA synthesis and PCR were performed through a continuous thermal*-*cycling program. Thermal-cycling program consists of four steps: Step one: one cycle of reverse transcription at 50 °C for 20 min; step two: one cycle of cDNA initial denaturation at 95 °C for 3 min; step three: 45 cycles consisting of two stages, a denaturation stage at 95 °C for 10 s and annealing, extension, and fluorescence measurement stage at 55 °C for 40 s; step four: one cycle of cooling at 25 °C for 10 s. According to the kit’s instructions, a positive reaction is detected by cycle threshold (Cq) values < 40 cycles, and PCR was repeated when the Cq values were between 40 and 45 cycles. In order to ensure that valid results were reported, negative and positive controls were run during each experiment. Moreover, the limit of detection for “Pishtaz Teb Zaman, Iran” was estimated at 200 copies/mL of sample. The diagnostic sensitivity and specificity of the used kit is 100%^1,25,26^.

### Identification of SARS-CoV-2 variants

**Figure 1:**
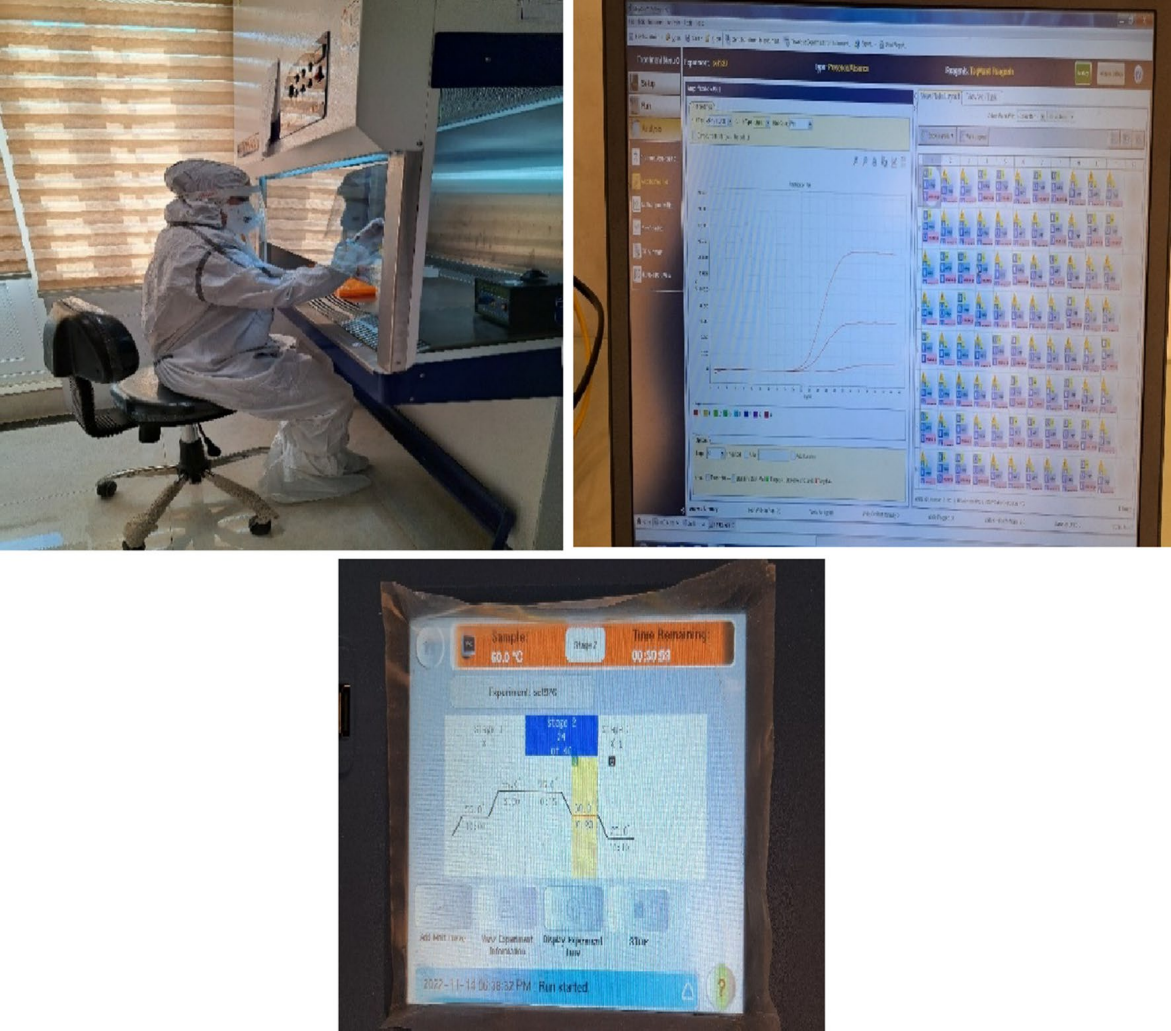
The illustration of experiments to detect of SARS-CoV-2 RNA genome in public environmental surfaces samples in the laboratory.

Due to the long-term impact of COVID-19 and emergence of more aggressive new variants, the progressive diagnosis of these variants can be used in diagnosis, infection prevention, and control plans. Qualitative TaqMan real-time PCR assay was used to determine the dominant variant of SARS-CoV-2. Geneva kit with a triple target gene was used to confirm Omicron and Delta variants. GA SARS-CoV-2 one-step RT-PCR testing kit covers three target genes specific to SARS-COV-2, N, S, ORF1a genes, and human RNase P gene. The sensitivity and specificity of the detection and differentiation of the two deletions in the S and ORF1a regions of the target kit have been reported as 100%. The limit of detection (LOD) for N gene was 100 copies/mL, it was 50 copies/mL for S gene, and 200 copies/mL of sample for ORF1a gene. Moreover, IPI SARS-CoV2 UK variant screening kit was used for detecting the UK variant by multiple spike protein mutations (deletion 69–70, deletion 144). The diagnostic sensitivity and specificity of this kit are 100%. The experiments performed in the laboratory for detecting SARS-CoV-2 RNA genome in the samples from public environmental surfaces are illustrated in Fig. 1.

### Ethical Approval

The authors would like to thanks Health and Environment research center, Ilam University of Medical Sciences, Ilam.

## Results and discussion

A total of 75 (21 positive environmental surface samples) contaminated environmental surfaces in different high- touch public places were examined, and the obtained findings as well as the detected positive/negative cases and sampling sites are presented in Tables 1, 2, 3 and 4. In the present research, on-site sampling was performed in different seasons (winter of 2021, summer of 2021, and winter of 2022) to assess the prevalence of the mentioned variants of COVID-19 in order to understand their capability to survive in different meteorological conditions and/or on different types of environmental surfaces. In addition, during the site sampling, the meteorological parameters including temperature (°C) and RH (%) were monitored, and the related results are demonstrated in Tables 1, 2, 3 and 4. The results of previous works on contaminated environmental/inanimate surfaces during the COVID-19 pandemic were compared with the results obtained in this work, and the findings are shown in Table 5.

**Table 1:** Detection of different variants of SARS-CoV-2 on frequently-touched environmental surfaces of different ATMs of Bank in various public places and seasons.

No.	Environmental surfaces/ seasonal sampling		Results	Cq-RdRp gene	Cq-N gene	Type of variants			Meteorological conditions	
						UK	Delta	Omicron	T (°C)	RH (%)
1	B1	**Winter 2021**	**Positive**	**35**	**33**				**13–27.5**	**18–67**
		**Summer 2021**	**Positive**	**33**	**34**		✓		**29.2–44.1**	**5–14**
		Winter 2022	Negative	–	–			✓	22.5–25.1	41–63
2	B2	Winter 2021	Negative	–	–	✓			13–27.5	18–67
		**Summer 2021**	**Positive**	**31**	**34**		✓		**29.2–44.1**	**5–14**
		Winter 2022	Negative	–	–			✓	22.5–25.1	41–63
3	B3	Winter 2021	Negative	–	–	✓			13–27.5	18–67
		Summer 2021	Negative	–	–		✓		29.2–44.1	5–14
		Winter 2022	Negative	–	–			✓	22.5–25.1	41–63
4	B4	**Winter 2021**	**Positive**	**31**	**29**	✓			**13–27.5**	**18–67**
		**Summer 2021**	**Positive**	**36**	**33**		✓		**29.2–44.1**	**5–14**
		Winter 2022	Negative	–	–			✓	22.5–25.1	41–63
5	B5	Winter 2021	Negative	–	–	✓			13–27.5	18–67
		**Summer 2021**	**Positive**	**34**	**36**		✓		**29.2–44.1**	**5–14**
		Winter 2022	Negative	–	–			✓	22.5–25.1	41–63
6	B6	**Winter 2021**	**Positive**	**30**	**28**	✓			**13–27.5**	**18–67**
		**Summer 2021**	**Positive**	**32**	**34**		✓		**29.2–44.1**	**5–14**
		Winter 2022	Negative	–	–			✓	22.5–25.1	41–63
7	B7	**Winter 2021**	**Positive**	**34**	**37**	✓			**13–27.5**	**18–67**
		Summer 2021	Negative	–	–		✓		29.2–44.1	5–14
		Winter 2022	Negative	–	–			✓	22.5–25.1	41–63
8	B8	**Winter 2021**	**Positive**	**32**	**35**	✓			**13–27.5**	**18–67**
		Summer 2021	Negative	–	–		✓		29.2–44.1	5–14
		Winter 2022	Negative	–	–			✓	22.5–25.1	41–63
9	B9	Winter 2021	Negative	–	–	✓			13–27.5	18–67
		Summer 2021	Negative	–	–		✓		29.2–44.1	5–14
		Winter 2022	Negative	–	–			✓	22.5–25.1	41–63
10	B10	Winter 2021	Negative	–	–	✓			13–27.5	18–67
		Summer 2021	Negative	–	–		✓		29.2–44.1	5–14
		Winter 2022	Negative	–	–			✓	22.5–25.1	41–63

*B1, B2, B3, B4,B5,B6,B7,B*,B9, and B10 are ATM of the Bank 1, 2, 3, 4,5,6,7,8,9 and 10 respectively in the different public places.

Significance values are [bold].

**Table 2:** Detection of different variants of SARS-CoV-2 on frequently-touched environmental surfaces of different POS Machine in various public places and seasons.

No.	Environmental surfaces/ seasonal sampling		Results	Cq-RdRp gene	Cq-N gene	Type of variants			Meteorological conditions	
						UK	Delta	Omicron	T (°C)	RH (%)
1	P1*	Winter 2021	Negative	–	–	✓			13–27.5	18–67
		Summer 2021	Negative	–	–		✓		29.2–44.1	5–14
		Winter 2022	Negative	–	–			✓	22.5–25.1	41–63
2	P2	Winter 2021	Negative	–	–	✓			13–27.5	18–67
		Summer 2021	Negative	–	–		✓		29.2–44.1	5–14
		Winter 2022	Negative	–	–			✓	22.5–25.1	41–63
3	P3	**Winter 2021**	**Positive**	**36**	**35**	✓			**13–27.5**	**18–67**
		Summer 2021	Negative	–	–		✓		29.2–44.1	5–14
		Winter 2022	Negative	–	–			✓	22.5–25.1	41–63
4	P4	Winter 2021	Negative	–	–	✓			13–27.5	18–67
		Summer 2021	Negative	–	–		✓		29.2–44.1	5–14
		Winter 2022	Negative	–	–			✓	22.5–25.1	41–63
5	P5	Winter 2021	Negative	–	–	✓			13–27.5	18–67
		Summer 2021	Negative	–	–		✓		29.2–44.1	5–14
		Winter 2022	Negative	–	–			✓	22.5–25.1	41–63

*P1, P2, P3, P4 and P5 are POS Machine 1, 2, 3, 4 and 5, respectively in the different public places.

Significance values are [bold].

**Table 3:** Detection of different variants of SARS-CoV-2 on frequently-touched environmental surfaces of different Gas station pump nozzles in various public places and seasons.

No.	Environmental surfaces/ seasonal sampling		Results	Cq-RdRp gene	Cq-N gene	Type of variants			Meteorological conditions	
						UK	Delta	Omicron	T (°C)	RH (%)
1	S1	Winter 2021	Negative	–	–	✓			13–27.5	18–67
		Summer 2021	Negative	–	–		✓		29.2–44.1	5–14
		Winter 2022	Negative	–	–			✓	22.5–25.1	41–63
2	S2	**Winter 2021**	**Positive**	**32**	**36**	✓			**13–27.5**	**18–67**
		**Summer 2021**	**Positive**	**36**	**37**		✓		**29.2–44.1**	**5–14**
		Winter 2022	Negative	–	–			✓	22.5–25.1	41–63
3	S3	Winter 2021	Negative	–	–	✓			13–27.5	18–67
		Summer 2021	Negative	–	–		✓		29.2–44.1	5–14
		Winter 2022	Negative	–	–			✓	22.5–25.1	41–63
4	S4	**Winter 2021**	**Positive**	**30**	**31**	✓			**13–27.5**	**18–67**
		**Summer 2021**	**Positive**	**32**	**34**		✓		**29.2–44.1**	**5–14**
		Winter 2022	Negative	–	–			✓	22.5–25.1	41–63
5	S5	Winter 2021	Negative	–	–	✓			13–27.5	18–67
		Summer 2021	Negative	–	–		✓		29.2–44.1	5–14
		Winter 2022	Negative	–	–			✓	22.5–25.1	41–63
6	S6	**Winter 2021**	**Positive**	**33**	**35**	✓			**13–27.5**	**18–67**
		Summer 2021	Negative	–	–		✓		29.2–44.1	5–14
		Winter 2022	Negative	–	–			✓	22.5–25.1	41–63
7	S7	Winter 2021	Negative	–	–	✓			13–27.5	18–67
		Summer 2021	Negative	–	–		✓		29.2–44.1	5–14
		Winter 2022	Negative	–	–			✓	22.5–25.1	41–63
8	S8	**Winter 2021**	**Positive**	**28**	**29**	✓			**13–27.5**	**18–67**
		**Summer 2021**	**Positive**	**35**	**34**		✓		**29.2–44.1**	**5–14**
		Winter 2022	Negative	–	–			✓	22.5–25.1	41–63

*S1, S2, S3, S4, S5, S6, S7 and S8 are Gas stations Pump Nozzle 1, 2, 3, 4, 5, 6, 7 and 8 respectively in the different public places.

Significance values are [bold].

**Table 4:** Detection of different variants of SARS-CoV-2 on frequently-touched environmental surfaces of different Escalator handrails of malls in various public places and seasons.

No.	Environmental surfaces/ seasonal sampling		Results	Cq-RdRp gene	Cq-N gene	Type of variants			Meteorological conditions	
						UK	Delta	Omicron	T (°C)	RH (%)
1	M1*	***Winter 2021***	***Positive***	***28***	***30***	✓			***13–27.5***	***18–67***
		Summer 2021	Negative	–	–		✓		29.2–44.1	5–14
		Winter 2022	Negative	–	–			✓	22.5–25.1	41–63
2	M2	***Winter 2021***	***Positive***	***32***	***34***	✓			***13–27.5***	***18–67***
		***Summer 2021***	***Positive***	***33***	***34***		✓		***29.2–44.1***	***5–14***
		Winter 2022	Negative	–	–			✓	22.5–25.1	41–63

* M1 and M2 are Mahestam Mall and Chahardah masoum Mall respectively in the different public places.

Significance values are [bold, italics].

In recent studies, it has been reported that using different molecular procedures, the SARS-CoV-2 RNA can be found in different contaminated environmental surfaces and facilities including hospital and non-hospital settings^1,9,14,27,28^. Although the evidence demonstrated that the highest detection rates of SARS-CoV-2 could be found in healthcare facilities and hospital settings with COVID-19 patients, other high-touch inanimate surfaces in public places can be important sources and transmission routes for COVID-19. This is due to the fact that most infected people are not aware of their disease, especially in the initial stages of the disease, and therefore spread the SARS-CoV-2 in public places^1,9,14,27,28^. Therefore, inanimate environmental surfaces can considered as one of the most important routes for SARS-CoV-2 transmission^17,18,18,19,29^.

Keeping this in mind, a total of 75 inanimate environmental surface samples were collected and examined for detection of the virus genome in three seasons (winters of 2021 and 2022, and summer of 2021), which are the same seasons that officials declared that different variants of SARS-CoV-2 were prevalent. This was carried out in order to survey the contamination of frequently-touched inanimate surfaces in public places. It is declared that in winter of 2021, the UK variant of concern (VOC) 202,012/01 (lineage B.1.1.7), in summer of 2021, the Delta (B.1.617.2) variant, and in winter of 2022, the Omicron variant were the predominant variants of SARS-CoV-2. The above-mentioned samples from inanimate surfaces in public places were taken from different locations including Bank ATMs, POS machines, gas station pump nozzles, and escalator handrails in malls.

The laboratory evidence confirmed that viable SARS-CoV-2 RNA could be detected on frequently-touched inanimate environmental surfaces, from 3 h in bioaerosols to 8–72 h on dry surfaces depending on the type of material of inanimate surfaces^8,18,30,31^. However, other studies reported that SARS-CoV-2 can survive up to 28 days on environmental and inanimate surfaces contingent upon characteristics of the environment and surface material. Therefore, this evidence confirmed that the viability of SARS-CoV-2 is higher and longer than other genera of coronaviruses including SARS-CoV-1 and MERS-CoV^6,8,18,32^.

The data related to detection of different variants of SARS-CoV-2 on frequently-touched environmental surfaces of different ATMs in various public places and seasons is demonstrated in Table 1. As it can be seen in Table 1, 10 out of 30 samples were positive and SARS-CoV-2 RNA was detected on different ATMs. The matter of survival of SARS-CoV-2 on different surfaces has been controversial in various studies (from hours to a few days). Riddell et al.^33^ showed that SARS-CoV-2 can be potentially infectious for longer than believed^33^. Van Doremalen et al. reported that SARS-CoV-2 stayed infectious in aerosols for hours, and up to days on different surfaces^31^. According to the studies, it has been determined that survival of SARS-CoV-2 in aerosols is about 3 h, and depending on the type of surface material, it can survive from hours to days. Moreover, apart from surface material, conditions such as temperature and humidity are effective on survival and infectiousness^7^. It is worth noting that since the surface material of ATMs is comprised of glass and plastic, it can be concluded that SARS-CoV-2 may survive longer on these types of environmental surfaces. On the other hand, most of the positive samples are related to the winter and summer of 2021, when the UK and Delta variants were dominant. Therefore, it can be concluded that these variants are probably more persistent on glass and plastic and are more resistant against temperature and RH. These results in accordance to Noorimotlagh et al.^18^.

The data related to detection of different variants of SARS-CoV-2 RNA on high-touch environmental surfaces of different POS machines in public places and seasons is demonstrated in Table 2. As it can be seen in Table 2, only one out of 15 environmental surface samples was positive and RNA of SARS-CoV-2 was detected. The one detected positive sample belonged to the winter of 2021 when the UK variant was predominant. It can be assumed that the contamination on this POS machine was low because only operators used it and it was not touched by other people.

Table 3 provides the data related to detection of different variants of SARS-CoV-2 RNA on frequently-touched environmental surfaces of different gas station pump nozzles in various public places and seasons. As it can be seen in Table 3, seven out of 24 surface samples were positive and SARS-CoV-2 RNA was detected in those locations. The positive samples were related to the winter and summer of 2021 when the UK and the Delta variants were predominant. Therefore, it can be concluded that these variants could persist in different meteorological conditions. The Delta variant persists in high temperatures (up to 44 °C). In a similar study, Karami et al.^34^ reported that five out of 36 inanimate surface samples (pump nozzle) were positive regarding the presence of SARS-CoV-2. However, they investigated the nozzle surfaces before and after the corona restrictions and after the quarantine, and all the samples were negative^34^. It is worth noting that the current study was performed after the quarantine conditions when different variants of COVID-19 were prevailing. Therefore, gas station pump nozzles could be a potential source of transmission of COVID-19. The results obtained herein are in agreement with other studies^8,10,14,35^.

Escalator handrails in malls are an important environmental surface for transmission of SARS-CoV-2. Therefore, the presence of SARS-CoV-2 on them was investigated as an essential and frequently-touched environmental surface in different public places and seasons, and the results are tabulated in Table 4. As it can be seen in Table 4, three environmental surface samples were positive in terms of the presence of SARS-CoV-2 RNA. The positive samples are associated with the winter of 2021 (when the UK variant was dominant) and the summer of 2021 (when the Delta variant was dominant). Therefore, it can be concluded that these variants might survive in different meteorological conditions (various temperatures and RH).

**Figure 2:**
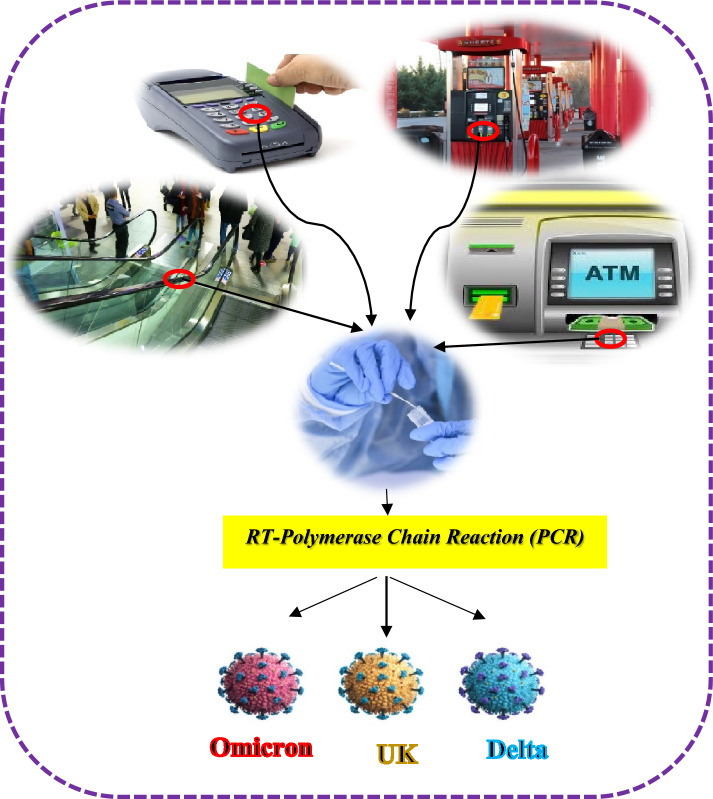
Schematic presentation of the environmental surfaces transmission chain of COVID-19 disease via public environmental surfaces.

The comparison of some similar investigations on detection of SARS-CoV-2 RNA on high-touch environmental surfaces of public places is demonstrated in Table 5. As illustrated in Table 5, most of the studies were carried out in healthcare settings and there are limited studies focusing on high-touch environmental surfaces in public places. The present study is one of the few studies focused on the spread of SARS-CoV-2 RNA on inanimate environmental surfaces, which have a great impact on the spread of COVID-19 in the world. As it can be seen in Tables 1, 2, 3 and 4, SARS-CoV-2 RNA was detected on different high-touch environmental surfaces of public places including bank ATMs, POS machines, gas station pump nozzles, and escalator handrails in malls, in various seasons with different meteorological conditions. The schematic presentation of the environmental surface transmission chain of COVID-19 via public environmental surfaces is presented in Fig. 2. Therefore, the results of this study suggested that preventive measures such as using suitable disinfectants in the desired duration are of utmost importance to prevent and limit the spread of COVID-19.

**Table 5:** Comparison of some similar investigations on the detection of SARS-CoV-2 RNA in the frequency-touched environmental surface of public places.

Author and year	Categories of samples	Country	Sampling site	Type of sample	sample size	Key findings (positive results) (%)
^35^	Surfaces of densely populated urban area	Brazil	Hospital care units and public squares	Public environmental surfaces	933	49 (5.25)
^29^	Health-care setting	South Korea	Environmental surface of 2 hospitals	Environmental surfaces	79	9 (16.45)
^1^	Health-care setting	Iran	Environmental surface of hospital	Environmental surfaces	76	40 (53)
^7^	Health-care setting	Iran	Environmental surface of hospital	Environmental surfaces	50	9 (18)
^36^	Health-care setting	China	Quarantine room during the incubation period	Environmental surfaces	50	17 (34)
^34^	public surfaces	Iran	Gas stations pump nozzle	Environmental surfaces	100	5 (5)
The present work	Public surfaces	Iran	ATM of banks, POS Machine, Gas stations pump nozzle and Escalator handrail of malls	Environmental surfaces	75	21 (28)

**All the previous studies using the sterile swab for sampling of environmental surface sampling and quickly transfer to the solution of viral transport medium (VTM).

## Conclusion

The present work focused on detection of SARS-CoV-2 RNA (genome) on high-touch public surfaces in different seasons, and was concerned with three variants (Delta, UK, and Omicron). A comprehensive sampling was performed and 75 samples were collected from different high-touch environmental surfaces in public places including bank ATMs, POS machines, gas station pump nozzles, and escalator handrails in malls and they were examined for the presence of SARS-CoV-2 RNA (genome), from which 21 (28%) samples were positive. Although the role of fomite transmission of COVID-19 is understood, more studies are required for determining the survival rate of the virus, and there should be continuous attempts to prevent the spread of SARS-CoV-2 including frequent cleaning and using efficient disinfectants on environmental surfaces, especially high-touch public places. In conclusion, the results of this study address the importance of the possibility of transmission by touching contaminated inanimate and environmental surfaces and they could be used to establish an effective protocol to prevent the indirect environmental transmission of SARS-CoV-2, interrupt the virus spread, and reduce the risk of infection.

## Data Availability

The datasets used and/or analyzed during the current study are available in the current form of manuscript.
